# Comparison of four lung scoring systems for the assessment of the pathological outcomes derived from *Actinobacillus pleuropneumoniae* experimental infections

**DOI:** 10.1186/1746-6148-10-165

**Published:** 2014-07-19

**Authors:** Marina Sibila, Virginia Aragón, Lorenzo Fraile, Joaquim Segalés

**Affiliations:** 1 Centre de Recerca en Sanitat Animal (CReSA), UAB-IRTA, Campus de la Universitat Autònoma de Barcelona, 08193 Bellaterra (Cerdanyola del Vallès), Spain; 2 Institut de Recerca i Tecnologia Agroalimentàries (IRTA), Barcelona, Spain; 3 Departament de Producció Animal, ETSEA, Universitat de Lleida, 25198 Lleida, Spain; 4 Departament de Sanitat i Anatomia Animals, Universitat Autònoma de Barcelona, 08193 Bellaterra (Cerdanyola del Vallès), Spain

**Keywords:** *Actinobacillus pleuropneumoniae*, Porcine pleuropneumonia, Experimental inoculation, Lung lesion scoring, Bacteriology, PCR

## Abstract

**Background:**

In this study, four lung lesion scoring methods (Slaughterhouse Pleurisy Evaluation System [SPES], Consolidation Lung Lesion Score [LLS], Image analyses [IA] and Ratio of lung weight/body weight [LW/BW]) were compared for the assessment of the different pathological outcomes derived from an *Actinobacillus pleuropneumoniae* (App) experimental infection model. Moreover, pathological data was coupled with clinical (fever, inappetence and clinical score), production (average daily weigh gain [ADWG]) and diagnostic (PCR, ELISA and bacterial isolation) parameters within the four infection outcomes (peracute, acute, subclinically infected and non-infected).

**Results:**

From the 61 inoculated animals, 9 were classified as peracute (presence of severe App-like clinical signs and lesions and sudden death or euthanasia shortly after inoculation), 31 as acutely affected (presence of App-like clinical signs and lesions and survival until the end of the experiment), 12 as subclinically infected (very mild or no clinical signs but App infection confirmed) and 9 as non-infected animals (lack of App-like clinical signs and lack of evidence of App infection). A significant correlation between all lung lesion scoring systems was found with the exception of SPES score versus LW/BW. SPES showed a statistically significant association with all clinical, production and diagnostic (with the exception of PCR detection of App in the tonsil) variables assessed. LLS and IA showed similar statistically significant associations as SPES, with the exception of seroconversion against App at necropsy. In contrast, LW/BW was statistically associated only with App isolation in lungs, presence of App-like lesions and ELISA OD values at necropsy.

**Conclusions:**

In conclusion, SPES, LLS and IA are economic, fast and easy-to-perform lung scoring methods that, in combination with different clinical and diagnostic parameters, allow the characterization of different outcomes after App infection.

## Background

*Actinobacillus pleuropneumoniae* (App) is the aetiological agent of porcine pleuropneumonia, a severe contagious disease distributed worldwide. This disease is characterized by haemorrhagic necrotizing pneumonia and fibrinous pleuritis, affecting mainly growing and finishing pigs [1]. The disease can take, even within a given batch of animals, three major clinical forms, namely peracute, acute or chronic disease [2]. These different disease presentations vary in severity depending on the age of the animals, the infecting App serovar and specific bacterial strain, environmental conditions, breeding genetic line susceptibility, pig immune status and magnitude of the exposure to the bacterium [1-4]. As a consequence, the clinical evolution of an App infection outbreak in a pig population might be very variable.

The main clinical signs observed in animals suffering from an acute App outbreak are high fever, vomiting, diarrhoea, anorexia and severe respiratory distress (increased respiratory rate, coughing/sneezing and dyspnoea) [1]. Animals with the peracute presentation may show all these clinical signs for a very short period of time, often overlooked under farm conditions, together with a foamy bloody nasal or oral discharge just prior death [2]. The animals that survive the acute phase of the disease may become chronically infected, showing little or no fever, mild coughing, inappetence and reluctance to move. Moreover, there is a proportion of animals that might remain infected without showing any apparent clinical sign. These subclinically infected animals are considered carriers of the infection [5].

Presence of animals with different disease presentations within a batch makes the App infection diagnosis challenging. In acute or peracute stages, presence of App-compatible clinical signs and/or lesions (haemorrhagic necrotizing pneumonia and fibrinous pleuritis) is usually sufficient to suspect from an App infection outbreak. However, other diagnostic techniques, such as serology, PCR or bacterial isolation, are needed for the detection of chronically or subclinically infected animals.

Presence and extension of App-compatible lesions can be assessed by visual estimation or using computed techniques (tomography, ultrasonography, sonography or radiography) [3,6]. Computed techniques allow the objective detection and quantification of lung lesions in dead but also in living animals [3]. Although these techniques can be excellent tools for experimental infections, their use at farm or slaughterhouse environments is limited because skilled personnel, specialized equipment and anesthetization of animals are required.

Thus, the objective of this study was to compare four economic and easy-to-perform lung lesion scoring methods for the assessment of the pathological outcomes derived from an App experimental infection. Moreover, these different outcomes were also characterized by means of clinical, productive and diagnostic parameters.

## Methods

### Animals

One hundred and fifteen11-week-old conventional male piglets were included in the study. The animals came from a farm free from App, *Mycoplasma hyopneumoniae (M. hyopneumoniae)* and porcine reproductive and respiratory syndrome virus (PRRSV). These piglets were used in 6 different App experimental inoculation trials performed at Centre de Recerca en Sanitat Animal (CReSA) Biosecurity Level 3 (BLS3) facilities. Experimental protocol, management conditions, animal inclusion criteria and personnel were the same in all 6 trials. Therefore, data coming from all the 6 trials was merged in the same database. Animal care and study procedures were conducted in accordance with the guidelines of Good Experimental Practice, under the approval of the Ethics Commission in Animal Experimentation of the *Generalitat de Catalunya* (Approved Protocol number 5796). Humane endpoints are described in the Additional file 1.

Before transportation to CReSA, selected animals were confirmed to be negative by serology against all App serovars (App Apx-IV Ab test, Idexx®) and *M. hyopneumoniae* (Blocking ELISA, *Mycoplasma hyopneumoniae* ELISA®, Oxoid, UK). Nasal swabs were tested by nested PCR (nPCR) to rule out *M. hyopneumoniae* infection [7].

### Inoculum preparation

App strain 4074 (SHOPE 4074, ATCC 27088; serovar 1, kindly donated by Dr. Marcelo Gottschalk, University of Montreal, Canada) was used to inoculate the animals. Bacterial inocula were prepared from freshly streaked chocolate-agar plates. After approximately 5 hours at 37°C and 5% CO_2_, the bacterial growth was resuspended in commercial PBS to give an optical density of 0.09 at 660 nm (this suspension corresponded to approximately 10^8^ CFU/mL). Bacterial concentration was confirmed by dilution of inoculum and plating on chocolate agar plates.

### Experimental design

Once at CReSA facilities, animals were weighed and randomly distributed into 2 groups based on their bodyweight. After one week of acclimatization, sixty one out of the 115 pigs (mean of 10 pigs per experiment) were intranasally challenged with a mean dose of 1.5x10^8^ CFU of App strain 4074 in 1.5 mL (half amount inoculated in each nostril). The remaining 54 (mean of 9 animals per experiment) pigs were inoculated with the same amount and by the same route with phosphate buffer saline (PBS). Control and App-inoculated animals were housed in different isolated pens. Animals were weighed and bled at challenge and necropsy days. After challenge, rectal temperature and clinical conditions were recorded twice per day. One week after challenge, animals were euthanized (Dolethal, Laboratorios Vetoquinol E.V.S.A.) At necropsy, App-like lesions such as fibrinous/fibrous pericarditis, pleuritis and lung abscesses/necrosis were evaluated. Moreover, lung lesions were scored using four different systems (see below). In addition, tonsilar and lung swabs and a portion of bronchial lymph node were collected from each animal. All the procedures conducted (weighting, sampling, clinical signs assessment and necropsy) were done always in the same order: firstly in control and secondly in challenged pigs. These samples were transferred to the laboratory, where they were processed for bacterial isolation (lung swabs) or PCR (tonsilar swab and bronchial lymph node).

### Clinical condition after challenge (clinical score) and innapetence

Animals were observed daily for respiratory effort and behaviour, and were scored as following: **score 0:** no signs of disease; **score 1:** increased breathing rate, occasional coughing and mild depression; **score 2:** abdominal breathing, usually lying down, standing when gentle stimulated; **score 3:** regular coughing or holding up on forelegs in a sitting position or markedly depressed or reluctant to stand up and with an increased heart rate (>110 beats per min); **score 4:** clinical score of 3 plus deteriorating towards showing signs of severe dyspnoea. Besides, appetite was daily scored as normal or abnormal.

### Rectal temperature

Rectal temperature was registered twice per day (at 7 am and 2 pm) from the challenge day onwards. Temperature measurements were done before any other manipulation. The evaluation of fever was established by means of a numerical score: 0 (less than 39.5°C), 1 (between 39.5 and 40.5°C) and 2 (higher than 40.5°C). This classification was modified from the one described by Moore et al. [8]. Animals scored 2 were considered to have an unequivocal raise in body temperature.

### Weight and average daily gain

Weight was recorded on challenge and necropsy days. Average daily gain (ADG) was calculated as the weight at necropsy minus the weight at challenge divided by the days lapsed between them.

### App lung lesion scoring systems

Four different lung lesion scores were calculated after the extraction of the lung from the thorax and after the removal of the larynx and heart.

#### ***Slaughterhouse Pleurisy Evaluation System (SPES)***

Presence of App-like lesions was scored 0 (no lesions), 1 (pleural fibrous/fibrinous adherences between cranio-ventral portions of cranial, medial and diaphragmatic lobes or monolateral mild adherences at the ventral margin of a diaphragmatic lobe), 2 (adherences with slight to moderate extensions into one of the diaphragmatic lobes), 3 (as score 2; but bilateral; in one of the diaphragmatic lobes can be extensive) and 4 (severely extended lesions, at least 1/3 of both diaphragmatic lobes) [9]. SPES is a subjective, fast and simple lung scoring system that provides information on the presence, extension, and localization of App-like lesions. This method is frequently used at slaughterhouse [9,10], where the use of other lung scoring system based on the schematic representation or photographing the lesions or weighting the lung is not feasible due to the speed or the structure of the slaughter chain.

#### ***Consolidation Lung Lesion Score (LLS)***

This method is based on the representation of the area showing lesions in a schematic map of the lung. In this scheme, each pulmonary lobe is divided by a number of triangles depending on the size of the lobe (7 for each cranial and middle lobe, 19 for each diaphragmatic lobe and 8 for the accessory one) [11]. The number of triangles affected with lesions per lobe is multiplied per five and divided by the number of triangles of each lobe (those lobes entirely affected with these lesions would have a score of 5). The maximum score of LLS is 35 (five points per lobe) [3]. LLS is the lung scoring system recommended by European Pharmacopoeia for testing *A. pleuropneumoniae* vaccines upon challenge with the bacterium [12]. This system is frequently used in experimental inoculations with different pathogens or in necropsy sessions, when time and space to represent the lung damage is available [13].

#### ***Image analyses (IA)***

IA is an objective method that allows the quantification of the affected lung area (%) by means of a picture and the use of the corresponding software (in this particular study, the ImageJ® online free software was used, http://rsbweb.nih.gov/ij). For such analysis, a picture was taken from the dorsal side of all studied lungs. The ventral side was pictured when the lesions were present only in that side. In order to obtain the percentage of affected lung, the area affected by lesions and total areas of the dorsal side of each lung were delimitated in each picture. All pictures were evaluated to the same bit scale. The percentage of affected area was obtained by the following formula:

$$\begin{array}{l} \left(\text{Right}\;\text{lung}\;\text{affected}\;\text{area}\;+\;\text{Left}\;\text{lung}\;\text{affected}\;\text{area}\right)/\\ \;\text{Total}\;\text{lung}\;\text{area}*100 \end{array}$$

This system allows having a photographic database of the lesions and to calculate the damaged lung surface when needed/desired. However, the need of a picture in the same position and the same bit scale makes its use at slaughterhouse very difficult.

#### ***Ratio of lung weight/body weight(LW/BW)***

This is an objective method that allows the quantification of the increased lung weight due to lesions with respect to the whole weight of the pig. This ratio was calculated using the following formula:

$$\left(\text{Weight}\;\text{of}\;\text{the}\;\text{lung}/\text{body}\;\text{weight}\right)*100.$$

### App re-isolation

Lung swabs were streaked on chocolate-agar plates in two sections in order to get isolated colonies. After overnight incubation at 37°C and 5% CO_2_, bacterial growth from samples from each side of the lung (right and left) was recorded and semi-quantified in the first section of the plate as: **0**, no growth; **1**, 1-19 colonies; **2**, 20-200 colonies; and **3**, confluent growth. The highest quantification of both sides of the lung was used to obtain the total bacterial score.

### App antibodies

Blood samples taken at challenge and necropsy were tested with a commercial ELISA kit (*Actinobacillus pleuropneumoniae* 1, 9, 11 Swinecheck® APP 1, 9, 11. Biovet. Canada).

### DNA extraction and App PCR detection

DNA from nasal and tonsilar swabs was extracted using Nucleospin blood, while DNA from bronchial lymph node was extracted with Nuclesopin tissues (both kits from Macherey-Nagel). DNA from both samples was tested by a nPCR that detects the APX IV gene, a toxin produced by all App serovars [14].

### Statistical analyses

All statistical analyses were carried out using the SAS system V.9.1.3 (SAS institute Inc, Cary, NC, USA). For all analyses, the individual pig was used as the experimental unit. The significance level (p) was set at 0.05 with statistical tendencies reported when p < 0.10. Presence of App lung lesions (assessed by the different lung scoring systems) and presence of App-like clinical signs were considered the primary and secondary experimental outcomes. The variables included in the statistical analyses were classified as dichotomous (mortality prior the end of the study, fever, clinical score higher than 0, inappetence, App re-isolation, presence of App-like lesions, seropositivity against 1, 9,11 App serovars, PCR in tonsil and PCR in bronchial lymph node), ordinal (maximum clinical score and SPES scoring system) or continuous (ADG, number of days showing fever, number of days showing inappetence, ELISA OD values at necropsy, LLS, IA and LW/BW). Shapiro Wilk’s and Levene tests were used to evaluate the normality of the distribution of the continuous variables and the homogeneity of variances, respectively. Two different statistical analyses were performed to test the: 1) association between the different variables with the four lung scoring systems, and 2) association among the four different lung scoring methods used. Contingency tables (Chi-square or Fischer exact tests) were used when the association between dichotomous and ordinal variables was assessed. To study the association between dichotomous or ordinal variables with the continuous non-normally distributed lung lesion scoring, the Wilcoxon test (with the U Mann-Whitney test to compare each pair of values) was used. To analyse the association between continuous normally distributed variables and dichotomous or ordinal variables, an ANOVA test (with Student’s T-test to compare each pair of values) was used. Finally, a linear or quadratic regression analysis was performed to evaluate the correlation between continuous variables.

## Results

### Clinical outcome

Control non-inoculated animals (n = 54) did not show fever, were clinically scored 0 through the whole study period and showed an ADG of 0.84 ± 0.20 Kg/d. In contrast, among the App inoculated animals, different severity of respiratory clinical signs (from 0 to 3) was observed. On one hand, nine animals showed peracute disease with fever, apathy, reluctance to move and (in five of them) foamy bloody nasal discharge. From these nine animals, five showed sudden death within the 24-36 h after the challenge and four were euthanized (within the same period) for animal welfare reasons. On the other hand, 39 animals survived until the end of the study with different severity of clinical signs and/or fever. Finally, 13 animals did not show either fever or any apparent clinical sign (score 0) through the whole study.

### Pathological outcome

Control animals did not show any lesions at necropsy. Lung lesions observed in the 9 animals that died shortly after inoculation consisted of unilateral or bilateral fibrino-haemorragic pleuropneumonia, with presence (in some of them) of foamy and bloody mucus exudate in the bronchi and trachea. In addition, 31 pigs showed unilateral or bilateral fibrino-to-fibrous necrotizing pleuropneumoniae and 21 animals did not show any App-like apparent lesions.

### Lung lesions scoring results

**Figure 1:**
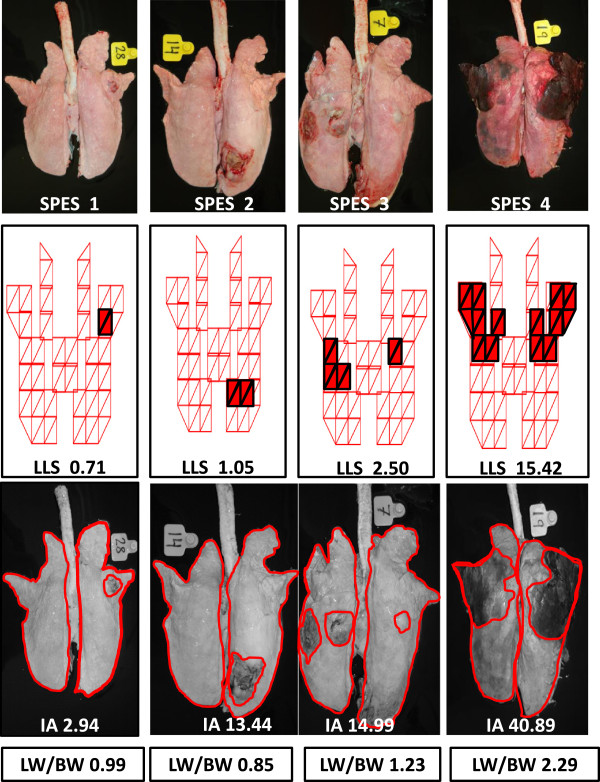
Examples of the lung lesions observed in animals intranasally inoculated with App serovar 1 and the corresponding value for the four lung lesions scoring systems used.

Examples of lung lesions derived from the App serovar 1 intranasal inoculation and the corresponding value of each scoring system used is given in Figure 1. Whereas SPES, LLS and IA were done in 61 animals, LW/BW was only performed in the 36 infected animals included in the last three trials. Reference values for this ratio LW/BW were provided by the control (non-inoculated) animals included in each of these three trials (n = 33). Ratio LW/BW was significantly higher in App inoculated animals (1.15 ± 0.48) than in their control counterparts (0.90 ± 0.202).

#### ***Relation between the four different lung scores with clinical variables***

**Figure 2:**
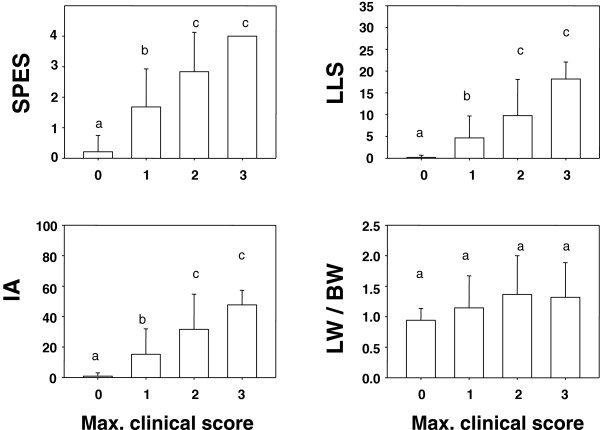
**Representation of the mean lung lesion score (±SD) regarding the maximum clinical score displayed during the experimental period.** SPES: Slaughterhouse Pleurisy Evaluation System; LLS: Consolidation Lung Lesion Score; IA: Image analyses; LW/BW: Ratio of lung weight/body weight. Different letter in superscript means statistically significant differences within a given variable (p < 0.05).

The relationship between each lung lesion score with the clinical dichotomous variables is detailed in Table 1. The relationship between the maximum clinical score with the four lung scoring systems is shown in Figure 2.

**Table 1:** Mean values and range (Max-Min) of each lung scoring system within each dichotomous clinical variable

**Lung score**	**Clinical variables**					
	**Mortality prior to the end of the study**		**Fever**		**Inappetence**	
	**Yes**	**No**	**Yes**	**No**	**Yes**	**No**
**SPES**	3.8^a^	1.2^b^	1.7^a^	0.1^b^	2.3^a^	0.7^b^
	(4-3)	(4-0)	(4-0)	(1-0)	(4-0)	(3-0)
**LLS**	17.6^a^	3.1^b^	6.9^a^	0.1^b^	8.1^a^	1.4^b^
	(31.4-10.7)	(20.9-0)	(31.5-0)	(1.4-0)	(31.5-0)	(7.7-0)
**IA**	53.3^a^	10.9^b^	22.3^a^	0.6^b^	26.2^a^	5.3^b^
	(88.4-34.1)	(54.5-0)	(88.4-0)	(5.9-0)	(88.4-0)	(32.3-0)
**LW/BW**	0.9^a^	1.1^a^	1.2^a^	1.0^a^	1.4^a*^	1.0^a*^
	(0.9-0.9)	(2.3-0.1)	(2.3-0.1)	(1.4-0.7)	(2.3-0.7)	(1.7-0.1)

SPES: Slaughterhouse Pleurisy Evaluation System; LLS: Consolidation Lung Lesion Score; IA: Image analyses; LW/BW: Ratio of lung weight/body weight. Different letter in superscript means statistically significant differences within a given clinical variable (p < 0.05).

*p = 0.07.

SPES, LLS and IA showed a statistically significant association with mortality prior to the end of the experiment, fever and inappetence (Table 1), as well as with maximum clinical score (Figure 2). In addition, these three scoring systems were also associated with the number of days that the animals showed fever, number of days with inappetence and number of days with clinical score higher than 0 (data not shown). In contrast, LW/BW showed only a statistical association with the number of days showing fever, number of days showing clinical score higher than 0 (data not shown) and a tendency with the presence of inappetence (Table 1) and the number of days with inappetence (data not shown).

#### ***Relation between the four different lung scores with the production variable ADG***

**Figure 3:**
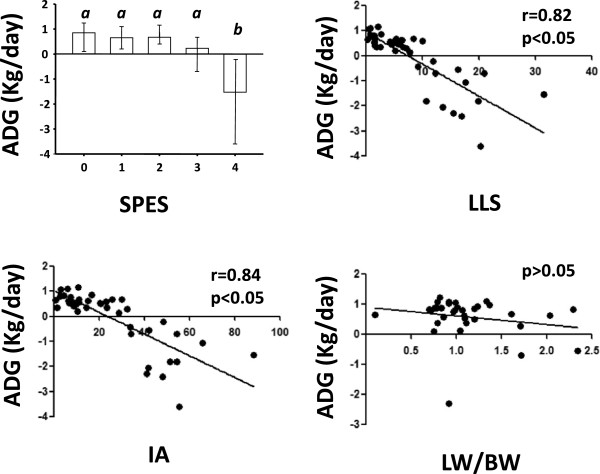
**Mean average daily weigh gain (ADG) (Kg/day ± SD) within each lung scoring system.** SPES: Slaughterhouse Pleurisy Evaluation System; LLS: Consolidation Lung Lesion Score; IA: Image analyses; LW/BW: Ratio of lung weight/body weight. Different letter in superscript means statistically significant differences within a given variable (p < 0.05).

SPES, LLS and IA showed a statistically significant association with ADG (Figure 3), while LW/BW did not.

#### ***Relation between the four different lung scoring and the diagnostic variables***

Relationship between each lung scoring and the different diagnostic dichotomous variables is detailed in Table 2.

**Table 2:** Mean values and range (Max-Min) of each lung scoring system within each of dichotomous diagnostic variable

**Lung Score**	**Diagnostic variables**									
	**App Re-isolation**		**Presence of App-like lesions**		**PCR in tonsilar swab**		**PCR in bronchial lymph node**		**Seropositivity to App 1,9,11 (at necropsy)**	
	**Yes**	**No**	**Yes**	**No**	**Yes**	**No**	**Yes**	**No**	**Yes**	**No**
SPES	2.3^a^	0.1^b^	2.5^a^	0^b^	1.8^a^	1.4^a^	2.3^a^	1.2^b^	1.9^a^	1.5^b^
	(4-0)	(1-0)	(4-0)		(4-0)	(4-0)	(4-0)	(4-0)	(4-0)	(4-0)
LLS	7.6^a^	0.1^b^	7.9^a^	0^b^	6.6^a*^	3.1^a*^	7.6^a^	3.8^b^	4.6^a^	5.5^a^
	(31.4-0)	(1.4-0)	(31.4-0.2)		(31.4-0)	(16.2-0)	(20.9-0)	(31.5-0)	(16.3-0)	(31.5-0)
IA	24.1^a^	0.2^b^	25.4^a^	0^b^	20.9^a*^	12.4^a*^	24.1^a^	13.2^b^	14.9^a^	18.5^a^
	(88.3-0)	(1.4-0)	(88.3-0.6)		(88.3-0)	(48.3-0)	(65.5-0)	(88.3-0)	(42.1-0)	(88.3-0)
LW/BW	1.2^a^	0.9^b^	1.3^a^	0.9^b^	1.1^a^	1.3^a^	1.3^a**^	1.02^a**^	1.0^a^	1.3^a^
	(2.3-0.1)	(1.3-0.7)	(2.3-0.1)	(1.4-0.7)	(2.0-0.1)	(2.3-0.7)	(2.3-0.01)	(2.3-0.7)	(2.3-0.8)	(2.3-0.1)

SPES: Slaughterhouse Pleurisy Evaluation System; LLS: Consolidation Lung Lesion Score; IA: Image analyses; LW/BW: Ratio of lung weight/body weight. Different letter in superscript means statistically significant differences within a given diagnostic variable (p < 0.05).

*p = 0.07; **p = 0.06.

SPES was statistically associated to all diagnostic variables but App PCR detection in tonsil. On the other hand, LLS and IA were statistically associated to all diagnostic variables but App PCR detection in tonsil (tendency) and seropositivity against App at necropsy. Finally, LW/BW was only statistically associated to App re-isolation and presence of App-like lesions. In addition, LW/BW showed a statistical tendency of association with App PCR detection in bronchial lymph node.

In addition, all the four lung lesion scores showed an association with the ELISA OD values of the sera obtained at necropsy (data not shown).

#### ***Correlation among the four different lung scores***

The correlation among the four lung scoring systems is detailed in Table 3. A significant correlation between all the lung lesion scoring systems was found with the exception of the correlation between SPES and LW/BW scores.

**Table 3:** Correlation between different lung scorings

**Lung scoring**	**LLS**	**IA**	**LW/LB**
**SPES**^**†**^	p < 0.05	p < 0.05	p > 0.05
	0 = 0^a^	0 = 0^a^	0 = 0.9 [1.4-0.7]^a^
	1 = 2.6 [10-0.3]^b^	1 = 7.0 [22.9-0.7]^b^	1 = 1.1 [2.3-0.1]^a^
	2 = 3.7 [6.4-1.1]^c^	2 = 13.1 [25.8-6.4]^c^	2 = 1.2 [2.0-1]^a^
	3 = 7.1 [12.2-2.5]^d^	3 = 26.2 [34.1-14.4] ^d^	3 = 1.3 [1.7-0.8]^a^
	4 = 17.00 [31.4-9.1]^e^	4 = 52.1 [88.4-33.7]^e^	4 = 1.7 [2.3-0.9]^a^
**LLS**	*	p < 0.05	p < 0.05
		r = 0.96	r = 0.46
		r^2^ value: 0.93	r^2^ value: 0.21
**IA**	*	*	p < 0.05
			r = 0.42
			r^2^ value:0.18

SPES: Slaughterhouse Pleurisy Evaluation System; LLS: Consolidation Lung Lesion Score; IA: Image analyses; LW/BW: Ratio of lung weight/body weight. ^†^Mean values and range [Max-Min] of lung scoring within each of the SPES categories. p values lower than 0.05 means that the pair of lung scoring system is significantly associated. Different letter in superscript means statistically significant differences within SPES categories.

### Characterization of the disease outcome

Evaluation of all the parameters described in the previous sections allowed the classification of the App inoculated animals into four categories according to the disease outcome displayed. A detailed description of the parameters used to establish such classification, as well as the statistical differences found between each category, is given in Table 4.

**Table 4:** Description of the different variables assessed in animals with different courses of disease

**Variable**		**Peracute**	**Acute**	**Subclinical**	**Uninfected**
		**(n = 9)**	**(n = 31)**	**(n = 12)**	**(n = 9)**
**Clinical signs**	Mortality prior to the end of the experiment (%)*	9 (100)^a^	0 (0)^b^	0(0)^b^	0 (0)^b^
	Fever at least one day (%)*	9 (100)^a^	29 (93.5)^a^	6 (50)^b^	2 (22.2)^b^
	Clinical score higher than 0 (%)*	9 (100)^a^	28 (90.3)^a^	2 (16.6)^b^	3 (33.3)^b^
	Mean clinical score (±SD)	1.88 (±0.80)^a^	1.32 (±0.70)^a^	0.16 (±0.4)^b^	0.44 (±0.8)^b^
	Inappetence at least one day (%)*	9 (100)^a^	21(67.7)^b^	3 (25.0)^c^	2 (22.2)^c^
**Production**	Mean ADG (±SD) (kg/d)	-1.91 (±0.83)^a^	0.47 (±0.4)^b^	0.83 (±0.2)^c^	0.9 (±0.2)^c^
**Diagnostic**	App-like lesions (%)*	9 (100)^a^	30 (96.7)^a^	0 (0)^b^	0 (0)^b^
	App isolation (%)*	9 (100)^a^	30 (96.7)^a^	3 (25)^b^	0 (0)^b^
	PCR detection in tonsil (%)*	8 (88.8)^a^	18 (58.0)^a^	10 (83.3)^a^	0 (0)^b^
	PCR detection in bronchial lymph node (%)*	5 (55.5)^a^	14 (45.1)^a^	4 (33.3)^a^	0 (0)^b^
	Seropositivity to App at death/necropsy*	0 (0)^a^	16 (51.6)^b,c^	3 (25.0)^a,c^	0 (0)^a^
	Mean SPES (Min-Max)	3.88 (3- 4)^a^	2.09 (1-4)^b^	0^c^	0^c^
	Mean LLS (Min-Max)	17.57 (10.71-31.4)^a^	5.17 (0.26-20.94)^b^	0^c^	0^c^
	Mean IA (±SD)	53.3 (±16.11)^a^	17.29 (±14.00)^b^	0^c^	0^c^
	Ratio LW/BW (±SD)	0.91(±0. 00)^a,b^**	1.27 (±0.54)^b^	0.88 (±0.21)^a^	0.96^a,b^ (±0.18)

Different letter in superscript means statistically significant differences within a given variable (p < 0.05). % = percentage; Min = minim; Max = Maximum; SD = standard deviation; *Number of animals showing or being positive by the corresponding parameter. **Data available only from one animal.

The 9 animals that did not survive until the end of the study were classified as peracutely affected animals. All these animals showed App gross lesions (uni or bilateral fibrino-necrotizing-haemorragic pleuropneumonia) and in all cases App was re-isolated from the lung. Whereas most of these animals were positive by PCR at tonsil (8/9) and/or at bronchial lymph node (5/9), all of them were seronegative against App at the moment of death. The remaining 52 pigs were classified as acutely affected (n = 31), subclinically infected (n = 12) and exposed but uninfected (n = 9) animals. Acutely affected animals were those that survived the initial phase of disease but showed evident clinical signs (dyspnoea and cough, fever, reduced appetite, and were reluctant to move). At the end of the study, these animals showed unilateral or bilateral fibrino-to-fibrous necrotizing pleuropneumoniae. All these animals (but one) were positive by culture and by PCR at one or two of the tested sites. From these 31 animals, 16 were seropositive against App 1,9,11 serovars at the moment of necropsy (7 dpi). Subclinically infected animals were defined as those in which App infection was confirmed (by re-isolation, PCR detection or antibody detection at necropsy) but showed little or no fever, mild or no clinical signs and did not show App-like lesions. Seven out of these 12 animals were seronegative and negative to App culture but positive by PCR either at the tonsil and/or at the bronchial lymph node. Finally, exposed but uninfected animals were those that were inoculated, but App was neither detected by PCR nor re-isolated, did not show either App clinical signs nor App-like lesions and no evidence of App seroconversion was observed at necropsy.

## Discussion

The present work aimed to compare 4 different scoring systems to evaluate lung lesions caused by App under experimental settings, and to correlate them with a number of clinical, productive and diagnostic parameters. Moreover, such complete characterization allowed classifying the animals in four different disease outcomes. In addition, this detailed characterization of App serotype 1 experimental inoculation outcome may help in reducing the number of animals used in future experimental trials (3Rs framework for conducting scientific animal experiments).

In general terms, a good correlation between all the lung lesion scoring systems (with the exception of SPES score versus LW/BW) was observed. SPES, LLS and IA showed a significant association with the majority of the variable examined (clinical, production and diagnostic variables). In contrast, LW/BW was statistically associated only with few variables. Thus, the LW/BW score was the least informative scoring method, most probably due to the unspecificity of the measure (weight of the lung, the large variability of the animals within each group and the low number of animals assessed). However, the fact that the ratio LW/BW was significantly higher in App inoculated animals than in their control counterparts, suggest that this measurement can be also of interest when the outcome of this disease is evaluated. In the literature, another lung scoring method based also on lung weight has been described [15]. In that system, the weight of the whole lung and the weight of the pneumonic lung tissue were registered. Probably this latter system provides much more precise information on the lung damage, but removal of lung tissue affected by App-like lesions in cases of pleuritis can be difficult.

Besides, the global analyses of the clinical, production and diagnostic (including the lung scoring systems) variables allowed expanding the knowledge on the different outcomes derived from an App inoculation. For example, it was observed that fever due to an App infection may appear when the App lung lesion score is low but may last up to five days in those animals with high lung lesions scores. In contrast, it was also noticed that not all the acutely affected animals showed fever. Moreover, inappetence was observed as the App lung lesions score increased. However, there were 10 acutely affected animals that did not show inappetence through the entire study. Under field conditions, these clinical signs (inappetence and fever) are difficult to assess since the follow up of individual animals is not practical. However, in such conditions, these clinical signs are probably observed for longer periods, especially in the acutely affected animals that do not die during the outbreak.

Evaluation of the respiratory distress and behaviour indicated a direct correlation between the clinical score and the App lung lesion score at necropsy. Nevertheless, there were five animals showing mild respiratory clinical scores (score 1 at one or two days after inoculation) that did not show any apparent App lung lesion at necropsy. While three of these animals were negative to App culture, PCR and serology (and therefore classified as exposed but uninfected), the other two were classified as subclinically infected pigs because they were positive by PCR but negative by serology and culture. This situation confirms the lack of specificity of the diagnoses based only on clinical signs, especially when these are mild [1].

Another interesting finding is that the SPES score 4 was observed in the lungs of animals affected by peracute, but also acute disease. Under field conditions, the peracutely affected animals would have probably died in a short period of time after showing clinical signs, suggesting an App outbreak. In contrast, these acutely affected animals, although suffered from important App lung lesions, would have probably developed a subsequent chronic App infection.

From a production parameter point of view, reduction of the ADG was only observed in peracute and acute cases of porcine pleuropneumonia. Indeed, in the present study, an App subclinical infection did not exert any effect on the ADG compared to the non-infected but exposed or non-challenged counterparts. However, it should be taken into account that animals were necropsied at 7 dpi and, therefore, results obtained in regards to ADG cannot be extrapolated to field conditions. In such situation, App infection may become chronic or subclinical, lasting for a longer period of time. In fact, in a previous field study, App subclinical infections resulted in a decrease of ADG of 30 g/d from nursery to slaughter [16].

In regards diagnostic variables assessed, a good correlation between presence of App lung lesions and App isolation from lung samples was obtained. Indeed, bacterial isolation from lungs is frequently used to confirm App involvement in acutely/peracutely affected animals, but should not be used in chronic infections since it can lead to false negative results [1]. In such chronic situations, in which necrosis is seen, the bacteria present in the lesions might be dead and therefore the isolation might be negative. This was probably the case of one acutely affected animal, in which App was not isolated from the observed lung lesions.

App subclinically infected animals (usually called App carriers) are usually identified by ELISA or PCR from tonsil samples [1,17]. In the present study, the percentage of subclinically infected pigs (no App-like lesions but PCR positive from tonsil samples) was higher than the percentage of seropositive animals. This is probably explained by the short time elapsed between inoculation and necropsy (maximum of 7 days), which may be also responsible for the lack of correlation between the lung lesion score and the seropositivity at the moment of necropsy. Nevertheless, seroconversion at one week post-infection has already been described using the same commercial ELISA kit [18].

At 7 dpi, the rate of App detection by PCR was higher in tonsils than in bronchial lymph nodes. App PCR in lymph nodes showed, however, a better correlation with lung lesion scores than that of tonsil. These results indicate that subclinically infected animals carry App only in tonsil (reservoir of the bacteria), while peracute and acutely affected animals, which develop lung lesions, have the bronchial lymph node also colonized by App.

In agreement with previously published studies, a high level of individual variation on the challenge outcome (ranging from fatal cases to uninfected animals) was observed [3,6,19-21]. Indeed, Tobias et al. [4] suggested that this individual variation may be caused by the non-homogeneous exposure to the pathogen when the intranasal inoculation is used. However, animals with different disease presentations have also been described in App experimental studies using aerosol [6,22] or intratracheal inoculation [23]. Another fact related with differences in the susceptibility to App infection of pigs is the breeding genetic line [24]. However, this would not be the case for the present study since, the same source of animals and the same breeding genetic line were used in the 6 different trials performed. Other differences linked to individual susceptibilities, which is a normal phenomenon in outbred animals, cannot be ruled out.

## Conclusions

In conclusion, intranasal inoculation of App serovar 1 led to different outcomes of disease. SPES showed a statistically significant association with all the clinical, production and diagnostic (but detection of App in the tonsil) variables assessed. LLS and IA showed the same statistically significant associations as SPES with the exception of seroconversion against App at necropsy. In contrast, LW/BW was statistically associated only with App isolation, presence of App-like lesions and ELISA OD values at necropsy. SPES, LLS and IA are economic, fast and easy-to-perform lung scoring methods that in combination with different clinical, productive and other diagnostic variables allowed the classification of such different App outcomes. Taking into account the difficulty to assess all these methods under practical conditions, SPES appears to be the most informative one.

## Competing interests

The authors declare that they do not have any competing interests.

## Authors’ contributions

VA prepared the inoculum and conducted the bacterial isolation. LF performed the statistical analyses. JS participated in the animal inoculation, necropsies and lesion scoring. MS designed the protocol, participated in the animal inoculation, blood sampling and necropsies and wrote the manuscript. All authors participated, revised and accepted the final version of the manuscript.

## Supplementary Material

Additional file 1 **Scoring system used to assess the animal clinical condition throughout the study.** Animals with one score of 3 or two scores of 2 in two different parameters were humanely euthanized immediately. Pigs with a score of 2 for the same parameter in two consecutive days were also euthanized. Click here for file
