# Supplementary material for: Comparison of four lung scoring systems for the assessment of the pathological outcomes derived from Actinobacillus pleuropneumoniae experimental infections
Source: BMC Vet Res. 2014 Jul 19;10:165. doi: 10.1186/1746-6148-10-165 (PMC4112831; doi:10.1186/1746-6148-10-165)
Supplement: Additional file 1 — Scoring system used to assess the animal clinical condition throughout the study. Animals with one score of 3 or two scores of 2 in two different parameters were humanely euthanized immediately. Pigs with a score of 2 for the same parameter in two consecutive days were also euthanized. [file 1746-6148-10-165-S1.docx]

**Additional File 1**

| **Score** | **Behaviour** | **Digestive signs** | **Respiratory signs** | **Locomotory signs** | **Nervous signs** |
| --- | --- | --- | --- | --- | --- |
| 0 | Active, playful, attentive | Clean hind legs, no diarrhoea observed | Normal | No locomotory signs | Normal |
| 1 | Sad, a bit passive | Dirty anus or pasty diarrhoea observed | Mild dyspnoea and/or coughing | Swelling of joint(s) visible, but none or only mild lameness. | Tremor or mild incoordination. |
| 2 | Mild depression, but still attentive | Dirty hind legs and/or watery diarrhoea observed | Severe dyspnoea and/or severe coughing | Swelling of joint(s) visible and/or moderate lameness. | Moderate nervous signs, difficulties to walk. |
| 3 | Severe depression, passive, prostrated. | Dirty hind legs with mucus and/or blood and/or severe diarrhoea with mucus or blood. | Abdominal breathing | Severe lameness, unwilling to move. | Severe nervous signs, unable to stand. |
